# 

**Published:** 1990-03

**Authors:** 


South West Paediatric Club

### WEST OF ENGLAND MEDICAL JOURNAL

**MARCH 1990**
**Volume 105(i)**

Copyright © West of England Medical Journal Co Ltd

**Contents**

1 **Contents**
2 **Editorials**
3 **Some Plymouth Worthies, part 1**
Michael Reilly
6 **Book Review**
The Health of Doctors, Clive Richards
7 **Coronaries, Cholesterol and Children**
June K. Lloyd
12 **Diet, Dermatology and Rotten Fish**
J. L. Burton
15 **The Prevention of Down's Syndrome in the South Western Region of England 1975–1985**
Nigel Wilson, Daisy Bickley, Alan McDermott
18 **Fats and Cancer**
D. E. Khoo, N. A. Habib
20 **Carcinoma of the Pancreas and Selectron Brachytherapy**
Michael Golby, C. Giles Rowland
22 **Brest Cancer Screening—an alternative view point**
Anthony Rodgers
24 **A new approach to Medical Records**
Denis Pereira Gray, Michael S. Hall
25 **Thyroxine. Osteoporosis and Fractures in the Mentally Handicapped**
J. Jancar
26 **From our Correspondents**—Monetarism and the General Practitioner, How to deal with the telephone sales menace, Traveller's Tale from You Nice States, Report on the VIII World Congress of Psychiatry, Athens
29 **Obituary**—Dr Gordon Heron
30 **South West Paediatric Club—Meeting at Barnstaple, May 1989**
34 **Wessex Branch of the Association of Clinical Pathologists, Meeting at Bristol, December 1988**

